# Repurposing α-Blockers Alfuzosin and Doxazosin for Cancer Therapy: Integrated Experimental, Mechanistic, and Molecular Docking Studies

**DOI:** 10.3390/ph19101519

**Published:** 2026-09-25

**Authors:** Mohammed M. Alanazi

**Affiliations:** Department of Pharmaceutical Chemistry, College of Pharmacy, King Saud University, Riyadh 11451, Saudi Arabia; mmalanazi@ksu.edu.sa

**Keywords:** drug repurposing, anticancer agents, α-adrenergic blockers, alfuzosin, doxazosin

## Abstract

**Background/Objectives**: Drug repurposing represents a promising strategy for determining new anticancer drugs through exploration of new therapeutic applications of clinically used drugs. Alfuzosin and doxazosin are α-adrenergic blockers clinically used for the treatment of benign prostatic hyperplasia (BPH). Growing evidence suggests that α-blockers may have anticancer properties. The purpose of this study was to investigate the potential of alfuzosin and doxazosin as repurposed anticancer medicines and to study their potential mechanisms of action, specifically their effects on apoptosis, cell cycle progression, enzyme panel, and cancer cell proliferation. **Methods**: The cytotoxicity of alfuzosin and doxazosin was investigated against multiple cancer cell lines using the MTT assay, with sunitinib used as a positive control. The enzyme inhibition effects of both drugs against potential target proteins were investigated, followed by cytometric analysis to determine the effects of the drugs on cell cycle progression and apoptosis in MCF-7 breast cancer cells. Molecular docking studies were also conducted to investigate possible interactions with the active site of EGFR. **Results**: Both alfuzosin and doxazosin demonstrated promising antiproliferative activity against cancer cells, with alfuzosin showing comparatively lower toxicity toward normal cells. The drugs also revealed potent inhibitory activity against the studied target proteins, pointing to their potential effect as multi-target inhibitors. Cell cycle analysis revealed that both drugs inhibited cell cycle progression at the G2/M phase in MCF-7 cells. Following treatment with alfuzosin and doxazosin, both drugs significantly induced apoptosis, with total apoptotic populations reaching 39.48% and 35.61%, respectively, compared with only 2.77% in untreated MCF-7 cells. Molecular docking studies further indicated the ability to interact with key amino acids within the EGFR active site. **Conclusions**: The present findings demonstrate the potential repurposing of alfuzosin and doxazosin as anticancer agents and offer a good background for further mechanistic and in vivo investigations.

## 1. Introduction

Cancer remains one of the leading causes of death worldwide despite notable progress in diagnosis and treatment. The Global Cancer Observatory (GLOBOCAN 2022) estimates that there were almost 20 million new instances of cancer and 9.7 million cancer-related deaths globally, and these figures are expected to rise significantly over the next 20 years due to population expansion and aging [1]. Treatment failure is still common because of intrinsic and acquired drug resistance, systemic toxicity, tumor heterogeneity, and the high cost of newly developed anticancer drugs, even though surgery, radiotherapy, chemotherapy, immunotherapy, and molecularly targeted therapies have greatly increased patient survival. As a result, developing safer and more effective treatment approaches remains a major priority in the treatment of cancer [2,3].

Tyrosine kinase inhibitors (TKIs), one type of targeted therapy, have completely changed the way cancer is treated by specifically blocking signaling pathways that control tumor growth, angiogenesis, migration, and survival. EGFR, HER2, VEGFR-2, PDGFR, SRC, and ABL are among the receptor and non-receptor tyrosine kinases whose dysregulation has been linked to the development and spread of many cancers. Despite the fact that over 80 TKIs have been used in clinical settings, kinase mutations, compensatory pathway activation, and tumor microenvironment modifications are common ways for resistance to arise. Thus, finding new tyrosine kinase inhibitors with better safety profiles continues to be a key goal in the search for anticancer drugs [4,5].

The process of creating a new anticancer medication is costly and time-consuming; it usually takes over ten years and billions of dollars, with a high rate of clinical failure development [6,7]. By finding new therapeutic uses for FDA-approved or clinically studied medications, drug repurposing—also known as drug repositioning—offers a compelling substitute. Compared to de novo drug discovery, these medicines’ clinical translation is significantly quicker and less expensive because they have proven pharmacokinetic, pharmacodynamic, and safety profiles. Consequently, one of the most promising methods for quickening the creation of specific cancer treatments is drug repurposing [8,9].

Through a variety of mechanisms, such as apoptosis induction, angiogenesis inhibition, epithelial–mesenchymal transition suppression, cancer metabolism modulation, immune response enhancement, inhibition of oncogenic signaling pathways, and tumor sensitization to targeted agents and conventional chemotherapy, repurposed drugs exert anticancer activities. Metformin, propranolol, itraconazole, mebendazole, disulfiram, and statins are among the non-oncology medications that have shown promising anticancer action in preclinical and clinical investigations, underscoring the translational potential of this approach [10,11,12].

The intrinsic anticancer effects of quinazoline-based α1-adrenergic receptor antagonists, which seem to be essentially independent of α1-adrenoceptor blockage, have drawn increased attention among repurposed therapeutic possibilities [13]. Clinically approved medications like doxazosin and alfuzosin (Figure 1) are frequently used to treat benign prostatic hyperplasia and hypertension. Both drugs are structurally members of the quinazoline family, a special scaffold that is widely found in tyrosine kinase inhibitors that have received clinical approval, including gefitinib, erlotinib, lapatinib, and afatinib. This pharmacophore implies that quinazoline α1-blockers might interact with kinase domains and serve as appealing scaffolds for the creation of new targeted anticancer drugs [14,15].

Quinazoline α1-blockers have been shown to have antiproliferative efficacy against a wide range of malignancies, including lung, pancreatic, prostate, breast, glioblastoma, renal, and colorectal cancers. These anticancer effects, in contrast to traditional α1-adrenoceptor antagonism, are mediated through a variety of molecular pathways, such as caspase-dependent activation of apoptosis, disruption of mitochondrial membrane potential, inhibition of the PI3K/Akt/mTOR pathway, suppression of NF-κB signaling, induction of autophagy, inhibition of angiogenesis, interference with focal adhesion kinase (FAK)-mediated cell adhesion, and reduction of metastatic potential. Together, these modulations reduce toxicity to normal cells while inhibiting the formation of tumors [11,15].

Doxazosin has attracted particular interest because it strengthens the effectiveness of EGFR-targeted therapy by overcoming osimertinib resistance through induction of autophagy and suppression of cancer stem cell survival, further reinforcing its potential for anticancer repurposing. On the other hand, alfuzosin has remained relatively underexplored; the presence of a quinazoline scaffold and its favorable pharmacological properties provide a rationale for investigating its potential anticancer activity and possible interactions with relevant molecular targets. From this perspective, repurposing alfuzosin and doxazosin represents an attractive strategy to exploit their established pharmacokinetic and safety profiles while exploring further interactions with molecular targets anticipated by Artificial Intelligence using SwissTargetPrediction, which is a web server for target prediction of small bioactive molecules. Proving potent anticancer activity of these well-known drugs would not only give a wide range of therapeutic applications but also enable discovery of lead compounds for the development of new targeted anticancer agents.

## 2. Results and Discussion

### 2.1. Potential Target Prediction

Comprehending the biological activities of bioactive compounds, anticipating possible adverse effects, and assisting in the optimization of therapeutic effects all depend on an understanding of the molecular mechanisms by which these compounds carry out their pharmacological actions [16,17]. A popular computational tool for predicting the protein targets of both known and unknown small compounds is the SwissTargetPrediction web service (http://www.swisstargetprediction.ch/ accessed on 16 June 2026). The strategy is predicated on the idea that substances with comparable chemical structures are probably going to interact with comparable biological targets. As a result, the platform identifies proteins linked to structurally related chemicals and compares the query molecule with known ligands to determine possible targets [18,19]. The chemical structures of alfuzosin and doxazosin were used to identify a number of proteins as potential targets using the SwissTargetPrediction online program. EGFR, VEGFR-2, Aurora kinase B and HDAC1 were the most predictable proteins linked to the development of cancer (Table 1). In order to determine the mechanism of action of alfuzosin and doxazosin as anticancer drugs, the inhibition assays of these targets were examined in vitro.

### 2.2. Antiproliferative Effects of Alfuzosin and Doxazosin on Multiple Cancer Cell Lines

A straightforward and popular technique for assessing a compound’s antiproliferative efficacy against cancer cells is the MTT assay. It quantifies the inhibitory effect of tested drugs by measuring cell viability based on the metabolic activity of living cells. The IC_50_ value, a helpful indicator of chemical potency, can also be computed using the assay. It is easier to determine whether the reported activity is specific to cancer cells rather than the result of generic cytotoxicity when a normal cell line is included. As a result, the MTT assay offers a crucial preliminary evaluation of the anticancer potential of recently investigated substances [20,21]. The lung tissue of a three-month-old female embryo was used to create the well-characterized normal human diploid fibroblast cell line WI-38. WI-38 is a valuable model for assessing the effects of anticancer drugs on non-malignant human cells since, in contrast to immortalized or tumor-derived cell lines, it maintains a normal diploid karyotype and has a limited replicative lifespan. It is often used as a normal-cell comparator in cytotoxicity experiments, such as those that directly compare WI-38 with altered and carcinoma cells to evaluate differential drug sensitivity. In order to evaluate the relative selectivity of alfuzosin and doxazosin toward cancer cells, WI-38 was used as a normal human reference cell line in this investigation [22,23,24].

The results in Table 2 demonstrate that both alfuzosin and doxazosin revealed cytotoxic effects against the cancer cell types under evaluation. Alfuzosin had modest activity, with fewer harmful effects on WI-38 cells and higher effects on some cancer cells, indicating moderate favorable selectivity toward the cancer-cell panel compared with WI-38 cells. Although doxazosin demonstrated greater cytotoxic activity, its impact on WI-38 cells should also be carefully taken into account when evaluating its overall therapeutic potential, as it does not show preferential selectivity against the investigated cancer cells; its cytotoxicity against WI-38 cells was actually higher than its average cytotoxicity toward the cancer cells. However, testing a single normal cell line is not sufficient evidence to provide broad normal-tissue selectivity, which needs further investigation using additional normal cell lines.

Finally, both drugs were less effective than sunitinib, the positive control, as was to be expected. However, these results imply that alfuzosin and doxazosin may have anticancer properties other than their established α-blocking efficacy. To further understand their modes of action, more investigations are required.

### 2.3. Inhibition Effects of Alfuzosin and Doxazosin on Multiple Enzymes

Results in Table 3 show that alfuzosin and doxazosin both exhibit inhibitory activity against the four targets under investigation—Aurora kinase B, EGFR, HDAC1, and VEGFR-2—according to the protein inhibition assays, however their potency varied depending on the target. Overall, alfuzosin had greater inhibitory activity against all four enzymes than doxazosin, indicating a more comprehensive and effective multi-target profile. Alfuzosin exhibited moderate inhibitory activity against Aurora kinase B, with an IC_50_ value of 0.28 ± 0.007 µm, while doxazosin was less effective (IC_50_ = 0.65 ± 0.014 µm). While alfuzosin’s activity stayed within the submicromolar range, both drugs were less active than the positive control danusertib (IC_50_ = 0.109 ± 0.003 µm). Since Aurora kinase B is crucial for cell division and mitotic progression, inhibition of this kinase may contribute to the antiproliferative actions of these substances [25]. Additionally, both medications demonstrated encouraging EGFR inhibition. With an IC_50_ value of 0.058 ± 0.0046 µm, alfuzosin was more powerful than doxazosin (0.092 ± 0.0033 µm). While erlotinib continued to be the most effective drug against EGFR (IC_50_ = 0.034 ± 0.001 µm), alfuzosin and doxazosin’s comparatively similar IC_50_ values show significant inhibitory action. Because EGFR signaling is often linked to cancer cell proliferation, survival, and tumor growth, this observation is especially pertinent. Previous results demonstrating doxazosin’s capacity to inhibit EGFR-related signaling pathways in cancer cells are likewise compatible with the observed EGFR inhibition. It is interesting to note that both compounds showed significant activity against HDAC1. Doxazosin’s IC_50_ was 0.231 ± 0.00064 µm, whereas alfuzosin’s was 0.105 ± 0.029 µm. Under the current assay conditions, both drugs were more powerful than the positive control entinostat (IC_50_ = 0.32 ± 0.0089 µm). Because HDAC1 inhibition can change gene expression and encourage cell-cycle arrest and apoptosis in cancer cells [26], this result is especially intriguing. Alfuzosin showed the greatest HDAC1 inhibitory activity of all the medications examined. Alfuzosin and doxazosin both had moderate inhibitory action against VEGFR-2, with IC_50_ values of 0.193 ± 0.0058 and 0.286 ± 0.0091 µm, respectively. Nevertheless, compared to the positive control (IC_50_ = 0.0162 ± 0.005 µm), their activities were significantly lower. However, given their possible multi-target anticancer efficacy, the submicromolar inhibition seen for both drugs may still have biological significance. Notably, earlier research has shown that doxazosin can reduce downstream angiogenesis-related pathways and interfere with VEGFR-2 signaling [27], which supports the current findings. Taken together, the findings show that alfuzosin has a more potent inhibitory effect against Aurora kinase B, EGFR, HDAC1, and VEGFR-2 than doxazosin. Both drugs demonstrated the highest efficacy against EGFR and HDAC1 among the studied targets, although VEGFR-2 exhibited less activity. The observed antiproliferative effects of these clinically used α_1_-adrenergic blockers may be explained mechanistically by their ability to simultaneously modulate several cancer-related pathways, which also supports their prospective repurposing as multi-target anticancer medicines.

Both alfuzosin and doxazosin showed comparatively strong inhibitory activity against the studied molecular targets in biochemical tests, but their antiproliferative effects in cancer cells were seen at much greater concentrations. When evaluating the pharmacological significance of these results, the apparent distinction between cellular activity and biochemical target inhibition is crucial. In intact cells, where drug activity may be influenced by cellular uptake, intracellular drug availability, protein binding, efflux mechanisms, metabolic stability, and the complex cellular environment, the lower concentrations needed to inhibit isolated molecular targets may not necessarily translate into comparable effects. Therefore, the enzyme inhibition findings should be considered supportive mechanistic evidence rather than direct evidence of therapeutic efficacy. Therefore, rather than being direct proof of therapeutic efficacy, the enzyme inhibition results should be viewed as supporting mechanistic evidence. While more pharmacokinetic, in vivo, and mechanistic research is needed to ascertain whether the concentrations linked to cellular activity are pharmacologically achievable, the current results offer preliminary preclinical evidence supporting further investigation of alfuzosin and doxazosin as potential anticancer repurposing candidates.

### 2.4. Cell Cycle Analysis of MCF-7 Cells Treated with Alfuzosin and Doxazosin

Since unchecked cell proliferation is a basic feature of cancer, cell cycle analysis is a crucial technique in the search for anticancer drugs. It is possible to determine whether an anticancer candidate suppresses cell proliferation by causing cell cycle arrest at particular checkpoints, such as G0/G1, S, or G2/M, by analyzing the distribution of cells across the various phases of the cell cycle. As a result, cell cycle analysis offers useful mechanistic data that supports cytotoxicity tests and aids in determining the possible mode of action of promising anticancer drugs [28].

As shown in Figure 2 and Table 4, both alfuzosin and doxazosin at their IC_50_ concentrations clearly changed the distribution of the cell cycle when compared to untreated cells, according to the cell cycle study of MCF-7 cells. The largest percentage of untreated MCF-7 cells were in the G0/G1 phase (57.28 ± 2.18%), followed by the S phase (32.59 ± 2.68%), and the G2/M phase (10.13 + 2.39%). Alfuzosin treatment significantly increased the G2/M population to 37.87 ± 6.91% while decreasing the G0/G1 population to 43.13 ± 4.46% and the S-phase population to 19.00 ± 6.63%. Similarly, doxazosin reduced the G0/G1 and S-phase populations to 39.91 ± 3.89% and 21.55 ± 3.93%, respectively, while causing a more noticeable increase in cells in the G2/M phase (38.54 ± 6.88%). These results imply that both drugs cause G2/M phase arrest in MCF-7 cells and obstruct normal cell cycle progression. Interestingly, doxazosin demonstrated a more potent impact than alfuzosin, suggesting a higher capacity to impede cell cycle progression at the G2/M checkpoint. This cell cycle arrest supports both drugs’ possible anticancer actions against cancer tumors and may be a factor in their antiproliferative activity.

### 2.5. Flow Cytometry Analysis of Induction of Apoptosis in MCF-7 Cells Treated with Alfuzosin and Doxazosin

Annexin V-FITC/PI flow cytometry was used to further examine the apoptotic effects of alfuzosin and doxazosin on MCF-7 breast cancer cells. Untreated MCF-7 cells showed a very low level of total apoptosis (2.99 ± 0.25%), as seen in Figure 3 and Table 5, suggesting the untreated cell population’s normal viability. Treatment with both drugs at their IC_50_ concentrations, however, significantly boosted apoptosis. Alfuzosin exhibited the greatest overall apoptotic impact (39.48 ± 1.72%), followed by doxazosin (35.61 ± 1.71%), indicating that both drugs may successfully cause MCF-7 cells to undergo programmed cell death. It is interesting to note that there were some variances between the two drugs’ apoptotic profiles. While doxazosin caused (7.97 ± 1.24%) early apoptosis and (22.17 ± 1.99%) late apoptosis, alfuzosin caused (11.31 ± 1.99%) early apoptosis and (22.99 ± 2.13%) late apoptosis. The prevalence of late apoptotic cells indicates that both drugs have a potent and long-lasting pro-apoptotic effect. Furthermore, the necrosis levels for doxazosin (5.47 ± 0.75%) and alfuzosin (5.18 ± 0.32%) suggest that apoptosis rather than nonspecific necrotic cell death was primarily responsible for the decrease in cell viability. Overall, these results corroborate the antiproliferative findings and imply that apoptosis induction plays a significant role in the anticancer action of doxazosin and alfuzosin against MCF-7 cells.

### 2.6. Molecular Docking of Alfuzosin and Doxazosin

Molecular docking is a powerful computational software in drug discovery that virtually visualizes a predicted binding between a ligand and the active site of a target protein and detects key interactions, such as hydrogen bonds and hydrophobic interactions. This analysis can help elucidate the potential pharmacological activity of investigated compounds [29]. EGFR was chosen as a representative model for molecular docking to deliver a structural hypothesis about the potential binding mode of alfuzosin and doxazosin. Docking validation was performed by calculating the RMSD of the aligned co-crystallized and redocked erlotinib (Figure S1). The RMSD of 1.746 Å demonstrated good alignment between the two poses.

The co-crystallized EGFR ligand erlotinib demonstrated characteristic binding within the EGFR active region (Figure 4). Additional hydrophobic interactions with residues including Leu694, Val702, Ala719, Leu820, and Leu764 helped stabilize the ligand within the binding pocket. It also formed significant hydrogen-bond interactions with residues in the ATP-binding pocket, especially around Met769. The robust and precise binding of erlotinib to EGFR is explained by these interactions.

On the other hand, doxazosin interacted with key amino acids in the EGFR binding pocket, including Lys721, Met769, Leu764, Val702, and Leu820, by making a combination of hydrogen-bond and hydrophobic interactions (Figure 5). Although its binding mode differed from that of erlotinib (Figure S2), the ability of doxazosin to occupy the same active-site region and interact with important residues supports its potential to interfere with EGFR activity. The capacity of doxazosin to occupy the same active-site region and interact with significant residues indicates its potential to disrupt EGFR activity.

Additionally, alfuzosin exhibited favorable interactions with the EGFR active site (Figure 6 and Figure S3). Crucially, the molecule interacted with Thr830 and Asp831 through hydrogen bonds, which could help to stabilize its orientation inside the binding pocket. Alfuzosin’s binding through hydrophobic and other non-covalent contacts was further supported by interactions with residues including Cys751, Val702, Leu820, Leu753, and Ala719. Overall, the docking results suggest that both alfuzosin and doxazosin can interact with the EGFR binding site, which indicates that the predicted interactions are consistent with the potent inhibition of the two drugs towards this protein. Table 6 summarizes the binding affinities, the principal hydrogen-bonding and hydrophobic interactions of alfuzosin, doxazosin, and erlotinib.

## 3. Materials and Methods

### 3.1. Materials

Sigma-Aldrich (St. Louis, MO, USA) provided the MTT assay kit (catalog No. CT02). BPS Bioscience (San Diego, CA, USA) provided the Aurora kinase B test kit (catalog No. 82094), EGFR assay kit (catalog No. 40321), HDAC1 assay kit (catalog No. 50061), and VEGFR-2 assay kit (catalog No. 40325). BioVision Technology, Inc. (Exon, PA, USA) sold the Annexin V-FITC kit (catalog No. K101-100). Abcam (Cambridge, MA, USA) supplied the Propidium Iodide Flow Cytometry Kit (catalog No. ab139418). The American Type Culture Collection (ATCC, Manassas, VA, USA) provided the cell lines utilized in this investigation via the Holding Company for Biological Products and Vaccines (VACSERA) in Cairo, Egypt.

### 3.2. Biological Evaluation

#### 3.2.1. Cytotoxicity Assay

A colorimetric MTT (3-(4,5-dimethylthiazol-2-yl)-2,5-diphenyltetrazolium bromide) test was used to measure cytotoxicity [30]. Cell viability, cytotoxicity, and proliferation are all commonly measured using the MTT assay. It is based on measuring mitochondrial activity by having living cells reduce MTT dye into formazan crystals [31]. The cells were seeded in RPMI-11,640 medium with 10% fetal bovine serum (FBS) and a combination of 100 L/mL streptomycin and 100 U/mL penicillin. Cancer cell lines were grown separately in 96-well plates at a density of 1.0 × 10^4^ cells/well, 37 °C, 5% CO_2_, and 100% relative humidity. The cells were incubated for 48 h before being treated with seven concentrations of sunitinib or the tested compounds ranging from 1.56 to 100 µm for 24 h. After applying 20 µL of MTT (5 mg/mL), the plates were incubated for four hours. The produced but insoluble purple formazan was then dissolved by adding 100 µL of DMSO to each well. Lastly, a BioTek Synergy H1 Plate Reader (BioTek Instruments, Inc, Winooski, VT, USA) was used to measure the generated color’s intensity at an absorbance of 570 nm. GraphPad Prism version 10.6.1 (GraphPad Software, San Diego, CA, USA) was used to fit the concentration-response curves and calculate IC_50_ values.

#### 3.2.2. Inhibition Assays of Enzymatic Activity

Tests for in vitro inhibition of enzymatic activity were compared to the tested protein kinase enzymes and HDAC1, as detailed in [32]. In summary, a specific human ELISA kit (Enzyme-Linked Immunosorbent Assay) was used to evaluate the inhibitory effectiveness of the tested compounds against Aurora kinase B, HDAC1, EGFR, and VEGVR-2. The specific antibody and enzymes were added individually to 96-well plates and left at room temperature for 2.5 h before 100 µL of the standard solution or the tested compounds were added (five concentrations ranging from 0.001 to 10 µm). The wells were cleaned after that. Next, each well was filled with 100 µL of the biotin antibody and allowed to sit at room temperature for one hour. Following additional washing, the wells were filled with 100 µL of streptavidin solution and allowed to sit at room temperature for 45 min. After a third washing step, 100 µL of TMB substrate reagent was added, and the plates were left for 30 min at room temperature. The color intensity was measured at 450 nm using the BioTek Synergy H1 Plate Reader (BioTek Instruments, Inc, Winooski, VT, USA) following the addition of 50 µL of the stop solution to each well.

#### 3.2.3. Cell Cycle Analysis

Using the methodology described in [33], the effect of the tested compounds on cell cycle distribution was investigated to determine the effect on the cell cycle progression of MCF-7 cells, while a flow cytometry study was carried out using a Propidium Iodide flow cytometry kit/BD. The cells were first grown at a density of 2 × 10^5^/well for 24 h. After that, the cells were exposed to the tested compounds for an entire day. The cells were then fixed with 70% ethanol for 12 h at 4 °C. The cells were then washed with cold PBS, treated with 100 µL of RNase A for 30 min at 37 °C, and stained with 400 µL of propidium iodide for 30 min at room temperature in the dark. The stained cells were identified using the Epics XLMCLTM flow cytometer (Beckman Colter, Apeldoorn, The Netherlands), and the results were assessed using Flowing software (version 2.5.1, Turku Center for Biotechnology, Turku, Finland).

#### 3.2.4. Flow Cytometry for Determination of Apoptosis

The Annexin V-FITC kit was used to assess the effect of the tested compounds on apoptosis, as detailed in [34]. MCF-7 cells were cultivated in 6-well plates at a density of 2 × 10^5^ and incubated for 24 h. The developed cells were then exposed to the tested compounds for an entire day. Following two PBS washes, the cells were trypsinized, collected by centrifugation (5 min, 300× *g*), and suspended in 0.1 mL of a 1X binding buffer. The cells were then double-stained with 5 µL Annexin V-FITC and 5 µL PI for 15 min at room temperature in the dark. An Epics XL-MCLTM Flow Cytometer (Beckman Colter, Apeldoorn, The Netherlands) was then used to examine the cells. The excitation wavelength was 488 nm, whereas the emission wavelength was 530 nm. The data was then analyzed using Flowing software (version 2.5.1, Turku Center for Biotechnology, Turku, Finland).

### 3.3. Molecular Docking

Alfuzosin, doxazosin and the ligand pose of erlotinib were docked into the active sites of EGFR using PyRx software equipped with AutoDock Vina (Version 0.8; The Scripps Research Institute, La Jolla, CA, USA). The RCSB protein data bank (https://www.rcsb.org/downloads accessed on 1 August 2026) provided the X-ray crystal structures of EGFR (PDB ID: 4HJO) for free. Discovery Studio Visualizer (v21.1.0.20298, Dassault Systèmes: San Diego, CA, USA) was used to visualize the results. Grid box (Center X:24.2698 Y:9.4307 Z:0.4984; Dimensions (Angstrom) X:14.6003 Y17:4711 Z:12.0835) was applied to reduce nonspecific binding and shorten the docking time. Exhaustiveness was set to 8, and generated poses were 0 to 8. The final pose selection was based on the alignment with the co-crystallized ligand (erlotinib). To create the protein crystal structures, all excess molecules, such as ligands, water, and sulfate, were first removed. The resulting data was saved in the PDB file format. After adding polar hydrogens to the previous PDB file, the data was then stored in PDBQT format. Third, using Discovery Studio Visualizer, the co-crystallized ligand was separated from the protein structure and stored in a PDB file. Doxazosin and alfuzosin were also drawn by ChemDraw Professional (version 16) and stored as PDB files. Doxazosin and alfuzosin were minimized and converted to PDBQT by PyRx, while the ligand pose was only converted to PDBQT by the same software. Finally, docking simulations were performed using PyRx, and the lowest-energy poses of doxazosin and alfuzosin were superimposed with the co-crystallized ligand to examine the interactions with the target proteins.

## 4. Conclusions

The present research provides preliminary evidence supporting the potential repurposing of the clinically used α-blockers alfuzosin and doxazosin as anticancer agents. Both drugs revealed significant cytotoxic activity against multiple cancer cell lines. Their capacity to inhibit specific protein kinases and HDAC1 provided additional evidence of their biological activity, indicating that kinase and HDAC1 regulation may be a factor in their anticancer actions. Both drugs significantly altered the progression of the cell cycle in MCF-7 breast cancer cells, accumulating cells in the G2/M phase. Additionally, doxazosin and alfuzosin successfully triggered apoptosis, suggesting that a key mechanism underpinning their antiproliferative action is programmed cell death. Molecular docking against EGFR provided a demonstrative structural hypothesis for the possible interactions of alfuzosin and doxazosin, supporting further investigation of their anticancer mechanisms. Collectively, the obtained results show that doxazosin and alfuzosin have untapped anticancer potential and encourage more research into them as potential treatment possibilities. To verify their effectiveness and possible clinical significance in cancer treatment, more mechanistic research and in vivo tests are necessary.

## Figures and Tables

**Figure 1:**
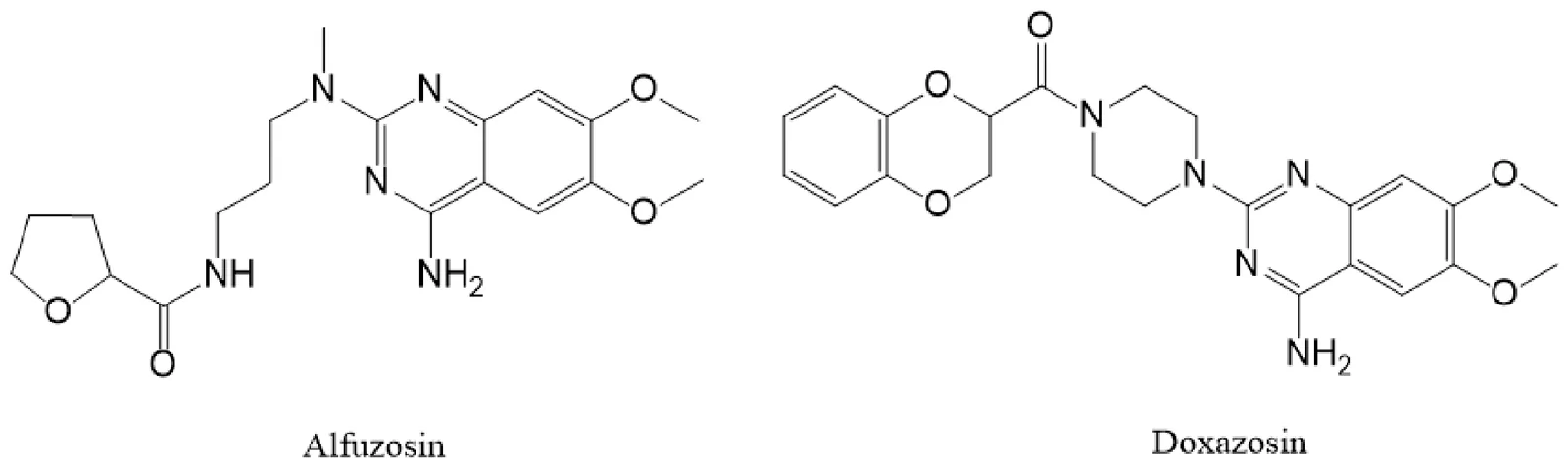
Chemical structures of alfuzosin and doxazosin.

**Figure 2:**
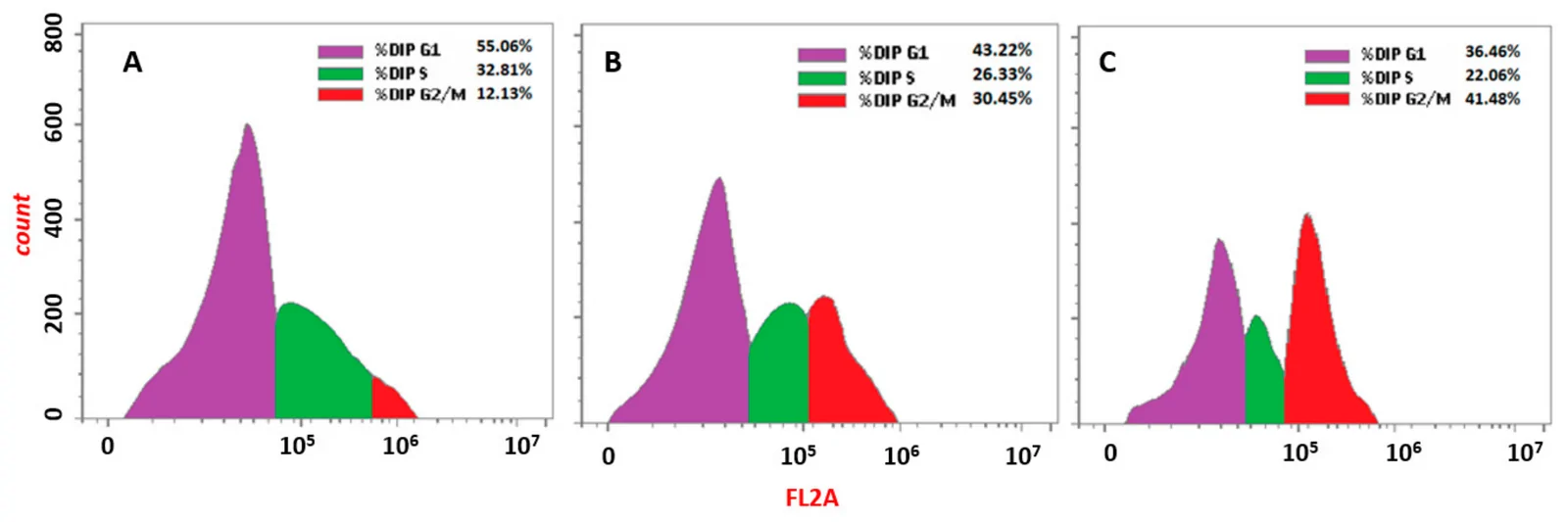
Cell cycle analysis of (**A**) untreated MCF-7 cells, (**B**) cells treated with alfuzosin and (**C**) cells treated with doxazosin. Figures are the most representative of three independent experiments.

**Figure 3:**
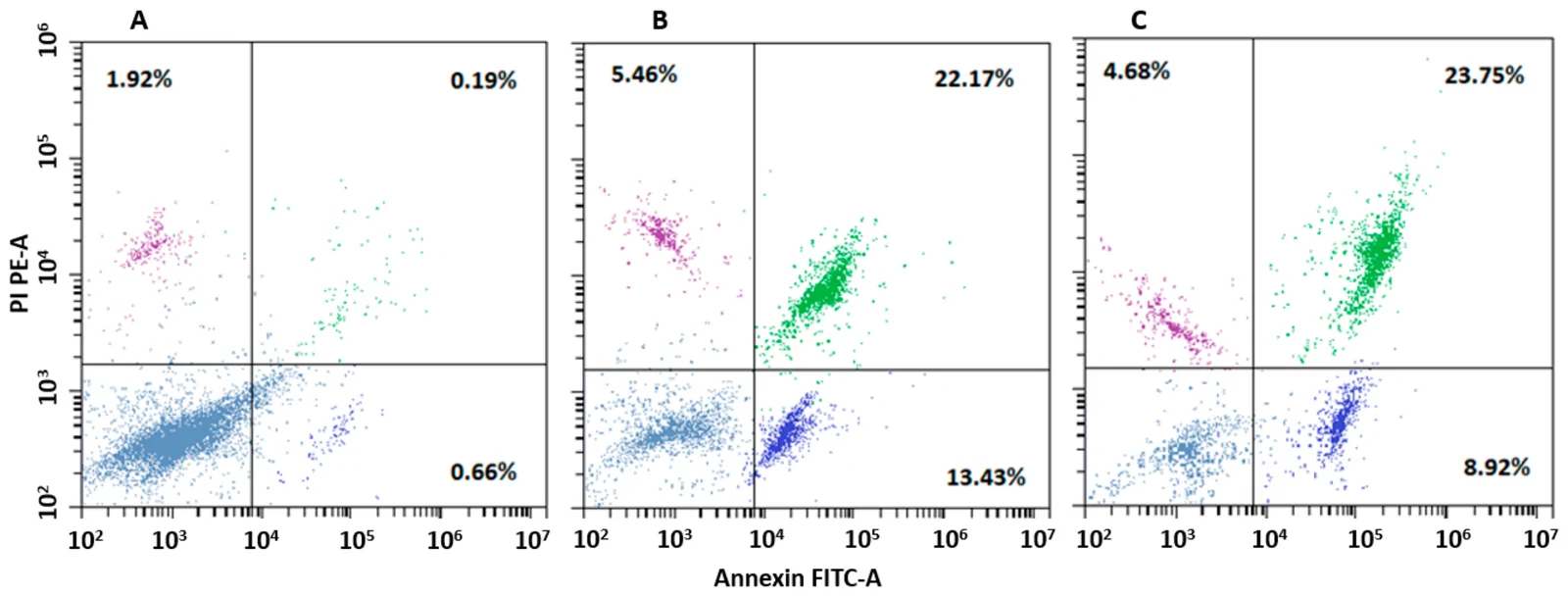
Flow cytometry analysis of (**A**) untreated MCF-7 cells, (**B**) cells treated with alfuzosin and (**C**) cells treated with doxazosin. Figures are the most representative of three independent experiments.

**Figure 4:**
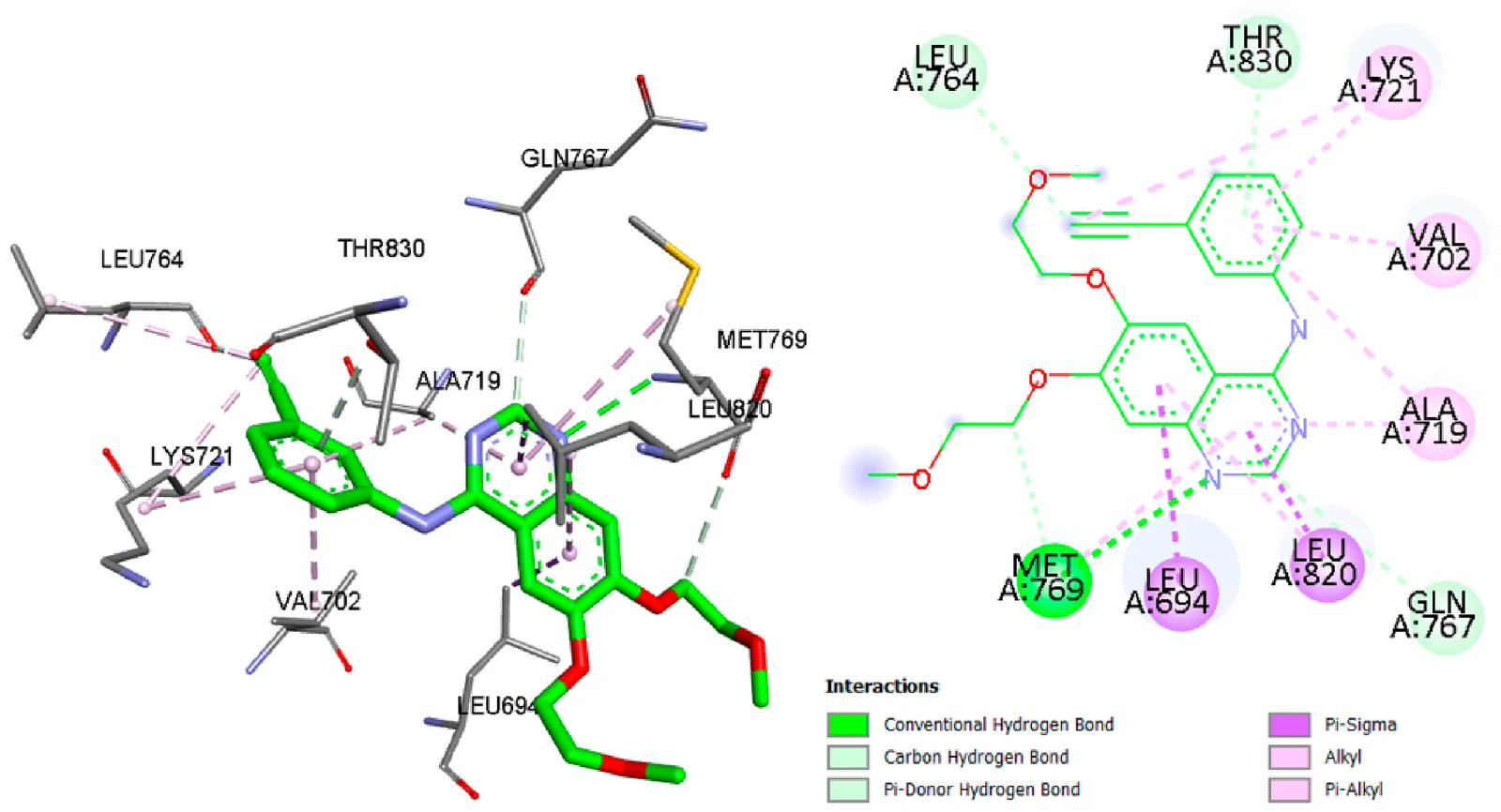
3d and 2d interactions of erlotinib with the active site of EGFR. Hydrogen bonds are labeled in green.

**Figure 5:**
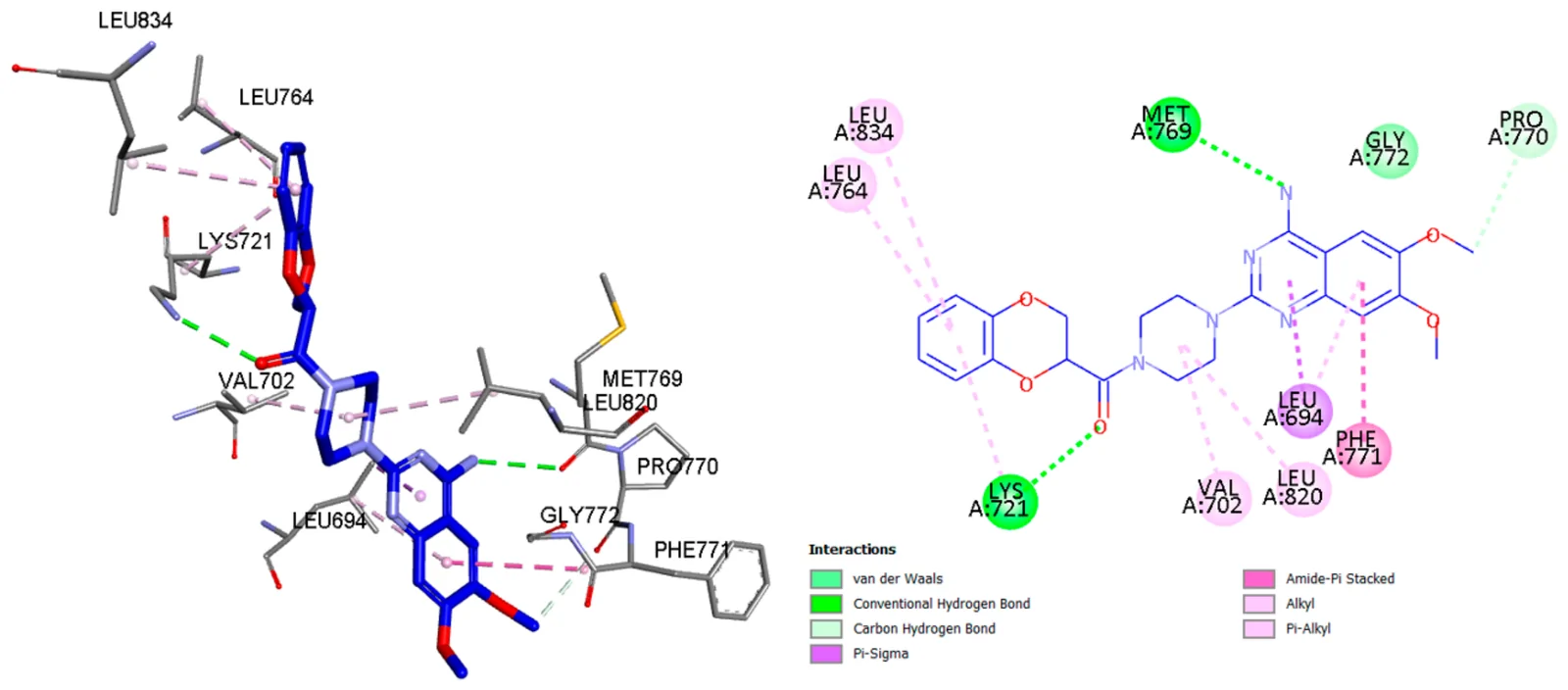
3d and 2d interactions of doxazosin with the active site of EGFR. Hydrogen bonds are labeled in green.

**Figure 6:**
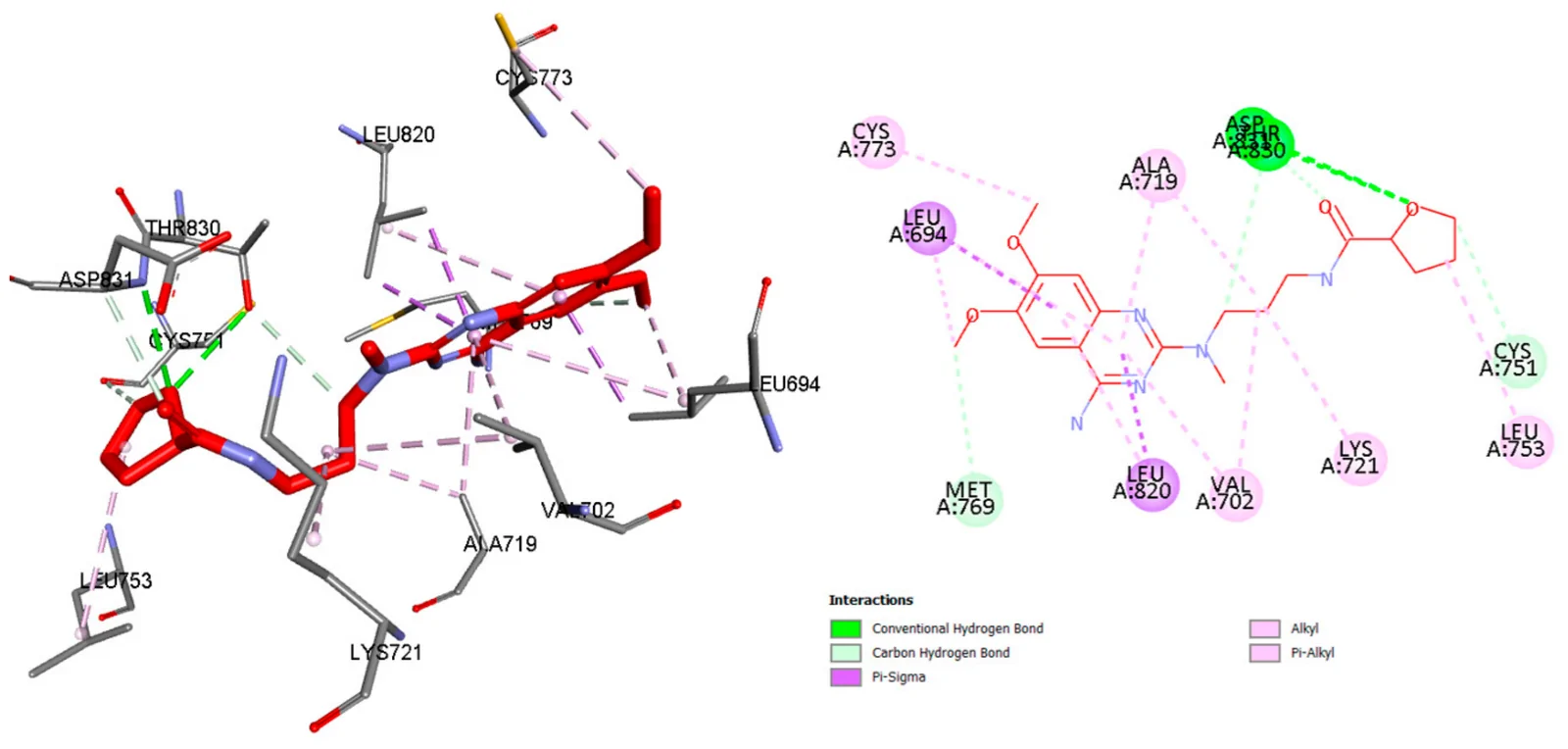
3d and 2d interactions of alfuzosin with the active site of EGFR. Hydrogen bonds are labeled in green.

**Table 1:** Target probabilities of predicted proteins for alfuzosin and doxazosin.

Drug Name	Target Probability			
	HDAC1	Aurora Kinase B	EGFR	VEGFR-2
Alfuzosin	0.217	0.045	0.057	0.069
Doxazosin	0.032	0.043	0.073	0.258

**Table 2:** Growth inhibition of selected cell lines induced by alfuzosin and doxazosin.

Drug Name	In Vitro Cytotoxicity IC_50_ (µm)					Selectivity Index (SI)
	Cancer Cells			Normal Cells		
	HCT-116	HepG-2	MCF-7	Ave. IC_50s_	WI-38	
Alfuzosin	69.24 ± 3.7	43.18 ± 2.5	38.01 ± 2.3	50.14	85.15 ± 4.3	1.7
Doxazosin	44.62 ± 2.6	31.53 ± 2.0	29.36 ± 1.9	35.17	18.91 ± 1.4	0.54
Sunitinib	11.75 ± 0.9	7.81 ± 0.5	5.92 ± 0.4	8.49	55.63 ± 3.2	6.55

**Table 3:** Inhibition effects of protein kinases and HDAC1 by alfuzosin and doxazosin.

Drug Name	IC_50_ (µm) of Enzymatic Activity			
	Aurora Kinase B	EGFR	HDAC1	VEGFR-2
Alfuzosin	0.280 ± 0.007	0.058 ± 0.0046	0.105 ± 0.0029	0.193 ± 0.0058
Doxazosin	0.650 ± 0.014	0.092 ± 0.0033	0.231 ± 0.0064	0.286 ± 0.0091
Danusertib	0.109 ± 0.003			
Erlotinib		0.034 ± 0.001		
Entinostat			0.32 ± 0.0089	0.0162 ± 0.005

**Table 4:** Cell cycle arrest of MCF-7 cells induced by alfuzosin and doxazosin.

Drug Name	DNA Content%		
	%G0-G1	%S	%G2/M
Alfuzosin/MCF-7	43.13 ± 4.46 *	19.00 ± 6.63	37.87 ± 6.91 *
Doxazosin/MCF-7	39.91 ± 3.89 *	21.55 ± 3.93 *	38.54 ± 6.88 *
Untreated MCF-7	57.28 ± 2.18	32.59 ± 2.68	10.13 + 2.39

All experiments were performed independently three times and data are presented as mean ± SD (*n* = 3). * *p* < 0.05 indicates statistical differences from the corresponding untreated MCF7 cells, as determined by unpaired *t*-test.

**Table 5:** Apoptotic cell distribution of MCF-7 cells treated with Alfuzosin and Doxazosin.

Drug Name	Apoptosis%			Necrosis
	Total	Early	Late	
Alfuzosin/MCF-7	39.48 ± 1.72 *	11.31 ± 1.99 *	22.99 ± 2.13 *	5.18 ± 0.32 *
Doxazosin/MCF-7	35.61 ± 1.71 *	7.97 ± 1.24 *	22.17 ± 1.99 *	5.47 ± 0.75 *
Untreated MCF-7	2.99 ± 0.25	0.77 ± 0.11	0.18 ± 0.04	2.04 ± 0.20

All experiments were performed independently three times and data are presented as mean ± SD (*n* = 3). * *p* < 0.05 indicates statistical differences from the corresponding untreated MCF7 cells, as determined by unpaired *t*-test.

**Table 6:** Summary table of the binding affinities, the principal hydrogen-bonding and hydrophobic interactions of alfuzosin, doxazosin, and erlotinib.

Drug Name	Binding Affinity (Kcal/Mole)	Hydrogen-Bonding	Hydrophobic Interactions
Erlotinib	−7	Met769	Leu694, Val702, Ala719, Leu820, and Leu764
Doxazosin	−9.4	Lys721 and Met769	Lys721, Met769, Leu764, Val702, and Leu820
Alfuzosin	−8.2	Thr830 and Asp831	Cys751, Val702, Leu820, Leu753, and Ala719

## Data Availability

The original contributions presented in this study are included in the article/Supplementary Materials. Further inquiries can be directed to the corresponding author.

## Supplementary Materials

The following supporting information can be downloaded at: https://www.mdpi.com/article/10.3390/ph19101519/s1. Figure S1: Alignment of co-crystallized erlotinib and redocked erlotinib; Figure S2: Alignment of erlotinib and doxazosin; Figure S3: Alignment of erlotinib and alfuzosin.
