# Supplementary material for: Repurposing α-Blockers Alfuzosin and Doxazosin for Cancer Therapy: Integrated Experimental, Mechanistic, and Molecular Docking Studies
Source: Pharmaceuticals (Basel). 2026 Sep 25;19(10):1519. doi: 10.3390/ph19101519 (PMC13708247; doi:10.3390/ph19101519)
Supplement: Supplementary file 1 [file pharmaceuticals-19-01519-s001.zip › pharmaceuticals-4565510-supplementary.pdf]

Supplementary information to:

Repurposing α-Blockers Alfuzosin and Doxazosin for Cancer Therapy: Integrated Experimental, Mechanistic, and Molecular Docking Studies

Mohammed M. Alanazi

Department of Pharmaceutical Chemistry, College of Pharmacy, King Saud University, Riyadh 11451, Saudi Arabia; mmalanazi@ksu.edu.sa

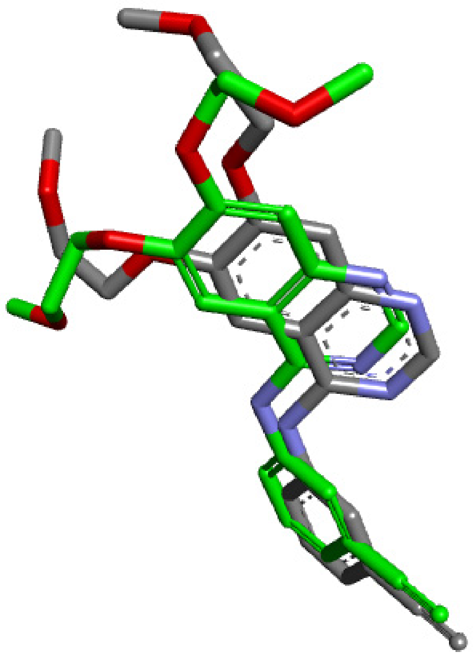

Figure S1: Alignment of co-crystalized erlotinib (green) and redocked erlotinib (gray).

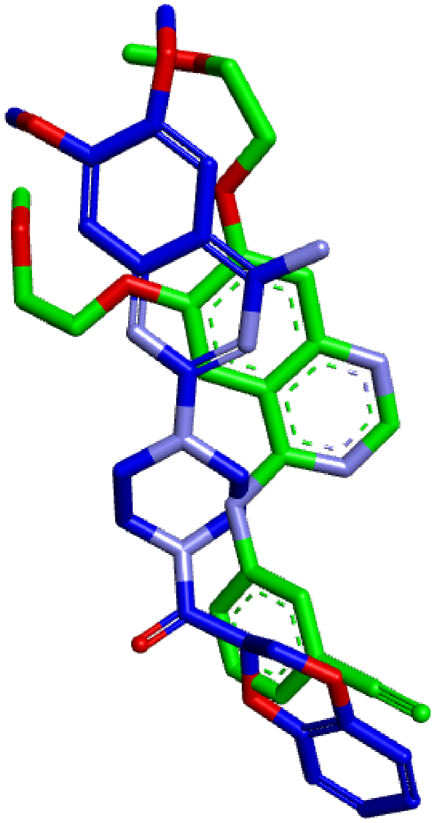

Figure S2: Alignment of erlotinib (green) and doxazosin (blue).

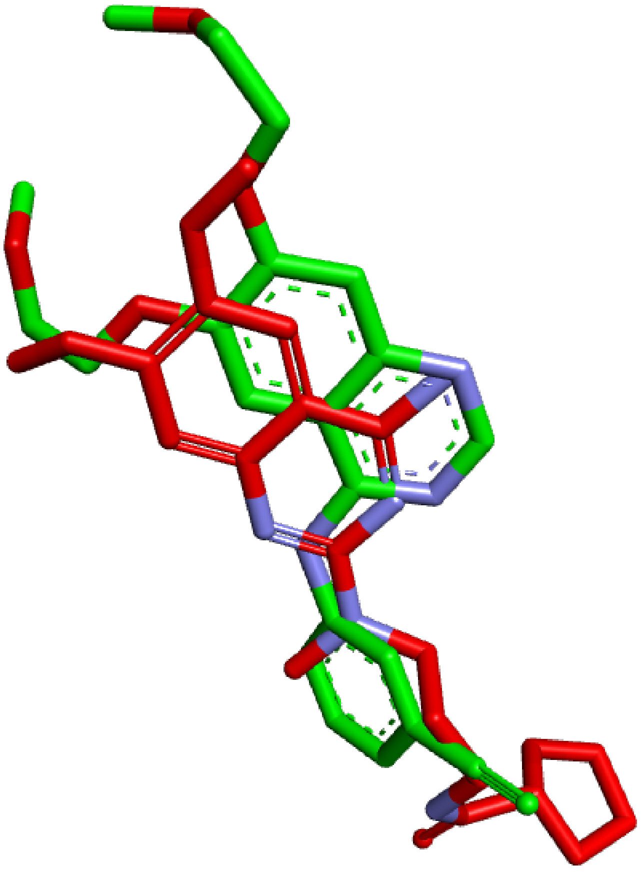

Figure S3: Alignment of erlotinib (green) and alfuzosin (red).
